# An effective approach for generating a three-Cys_2_His_2 _zinc-finger-DNA complex model by docking

**DOI:** 10.1186/1471-2105-11-334

**Published:** 2010-06-18

**Authors:** Chun-Chi Chou, M Rajasekaran, Chinpan Chen

**Affiliations:** 1 Graduate Institute of Life Sciences, National Defense Medical Center, Taipei 114, Taiwan; 2 Institute of Biomedical Sciences, Academia Sinica, Taipei 115, Taiwan; 3 Department of Life Science, National Tsing Hua University, Hsinchu 300, Taiwan; 4 Chemical Biology and Molecular Biophysics, Taiwan International Graduate Program, Institute of Biological Chemistry, Academia Sinica, Taipei 115, Taiwan

## Abstract

**Background:**

Determination of protein-DNA complex structures with both NMR and X-ray crystallography remains challenging in many cases. High Ambiguity-Driven DOCKing (HADDOCK) is an information-driven docking program that has been used to successfully model many protein-DNA complexes. However, a protein-DNA complex model whereby the protein wraps around DNA has not been reported. Defining the ambiguous interaction restraints for the classical three-Cys_2_His_2 _zinc-finger proteins that wrap around DNA is critical because of the complicated binding geometry. In this study, we generated a Zif268-DNA complex model using three different sets of ambiguous interaction restraints (AIRs) to study the effect of the geometric distribution on the docking and used this approach to generate a newly reported Sp1-DNA complex model.

**Results:**

The complex models we generated on the basis of two AIRs with a good geometric distribution in each domain are reasonable in terms of the number of models with wrap-around conformation, interface root mean square deviation, AIR energy and fraction native contacts. We derived the modeling approach for generating a three-Cys_2_His_2 _zinc-finger-DNA complex model according to the results of docking studies using the Zif268-DNA and other three crystal complex structures. Furthermore, the Sp1-DNA complex model was calculated with this approach, and the interactions between Sp1 and DNA are in good agreement with those previously reported.

**Conclusions:**

Our docking data demonstrate that two AIRs with a reasonable geometric distribution in each of the three-Cys_2_His_2 _zinc-finger domains are sufficient to generate an accurate complex model with protein wrapping around DNA. This approach is efficient for generating a zinc-finger protein-DNA complex model for unknown complex structures in which the protein wraps around DNA. We provide a flowchart showing the detailed procedures of this approach.

## Background

Determining the structure of protein-DNA complexes and elucidating the details that govern their interaction is essential to better understand many biological processes. In many instances, limitations in crystallization and difficulties in obtaining the intermolecular nuclear Overhauser effects by NMR experiments are obstacles to determining the structure of protein-DNA complexes [1]. Homology modeling is an alternative approach to obtain a protein-DNA complex model. Programs such as TFmodeller can model the complex according to homologous complex structure [2]. The major limitation of this approach is that high conservation of interface residues between the target and template is required for generating a good homology complex model. The high conservation of interface residues may not be possible in many cases; for example, in the zinc finger protein family, the DNA recognition residues and the interacting DNA are not well conserved. Thus, the prediction of the detailed interaction for the entire zinc-finger protein-DNA complex based on the homologous complex structure may not be effective. Hence, other approaches are required to obtain good complex models.

Few structurally based approaches to understand and predict the specificity and binding affinity of the zinc-finger protein-DNA interactions have been reported [3-5]. The applicability of these structurally based approaches will significantly increase with the availability of zinc-finger protein-DNA complex models. One study [6] used homology models to predict the binding affinities and specificities of protein-DNA complexes, including zinc-finger-DNA complexes. However, the homology modeling complexes are limited by sensitivity to protein and DNA backbone orientation [7], which may affect the prediction of the detailed interaction between the protein and DNA.

Biomolecular docking is an alternative approach to modeling zinc finger protein-DNA complexes. However, the inherent flexibility of DNA and the scarcity of information about the precise surfaces of DNA involved in interactions with associated proteins represent two major hurdles in computational docking [8]. High Ambiguity-Driven biomolecular DOCKing (HADDOCK) [9] is an information-driven program that successfully addresses the global and local DNA flexibility in modeling protein-DNA complexes. The information on interfaces is derived from biochemical and/or biophysical experiments and introduced as ambiguous interaction restraints (AIRs) [10] to drive the protein-DNA docking. Although several studies have successfully used HADDOCK in generating protein-DNA complex models [11-15], none have analyzed the proteins that wrap around the DNA, such as the three-Cys_2_His_2 _zinc-finger-DNA complex. In this study, we focused on modeling the entire three-Cys_2_His_2 _zinc-finger-DNA complex by use of the HADDOCK program.

For protein-DNA complexes, two structural factors determine binding geometries: the tight fitting between DNA and protein surfaces and the matching of the residue and base positions [16]. Several challenges must be factored into generating a model of the three-Cys_2_His_2 _zinc-finger-DNA complex with the HADDOCK program, including the number and position of AIRs and the combination of active residues and bases of AIRs in rigid body docking. However, the combination of active residues and bases of AIRs in the multiple DNA binding domains results in more complexity. In this study, we focused on the number and position of AIRs and simplified the combination of active residues by defining the AIRs in a pairwise manner between amino acids and bases. This approach mainly limits the combinational search, and, hence, the overall geometric distribution of AIRs between domains depends on the number and position of AIRs in the interface.

Here, we used the Zif268-DNA complex structure [17] as a reference system for docking. From the interaction information for this complex structure, three different AIR sets were derived and used for docking calculation. The docking result for each AIR set was evaluated for the total number of wrap-around conformations, interface RMSD (iRMSD), buried surface area (BSA), and fraction native contacts (F_nat_) of the modeled complex. We found that the third AIR set was sufficient to generate good complex models for Zif268-DNA, and the same method was then used to model other zinc-finger protein-DNA models, such as YY1 [18], WT1 [19] and Aart [20], by using only two AIRs in each domain, that is, the third AIR set. Thus, the three-Cys_2_His_2 _zinc-finger-DNA complex models could be successfully generated by using only two AIRs in each domain and the HADDOCK program.

We then extended this method to model the unknown Sp1-DNA complex structure. The human transcription factor Sp1 is considered a ubiquitous factor that regulates the expression of different genes responsible for various cellular processes [21-23]. The C-terminal DNA binding domain of Sp1, referred to as Sp1 hereafter, consists of three consecutive Cys_2_His_2 _zinc fingers that bind to GC-rich recognition elements present in a number of cellular and viral promoters. To date, the structure and computational model of Sp1-DNA have not been reported. However, Oka *et al *[24] reported the binding mode and proposed detailed interactions between Sp1 and DNA on the basis of similarity of their Sp1 NMR structure with the Zif268 protein structure. The reported binding mode is in good agreement with results of other experiments such as ethylation interference analysis [25], methylation interference analysis and mutation study [26]. In this study, we built the homology structure of Sp1 and then used the reported interactions to derive two AIRs in each finger domain to generate the Sp1-DNA complex model. The interactions observed on the best Sp1-DNA complex model are in good agreement with those previously reported [24], which further reveals that the approach we developed is indeed an efficient way for generating a zinc-finger protein-DNA complex model in which the protein wraps around DNA.

## Results and Discussion

### Overview of the docking approach

First, we give a brief overview of the data-driven docking for generating a three-Cys_2_His_2 _zinc-finger-DNA complex. Using the X-ray crystal structure of the classical three-Cys_2_His_2 _zinc-finger Zif268-DNA complex as a reference, we obtained detailed information on hydrogen bonds and van der Waals contacts between Zif268 and DNA [27]. From this information, we evaluated three different AIR sets for generating complex models using the HADDOCK program.

The first set was derived from the complete interface information on hydrogen bonds and van der Waals contacts, and the second set was derived from information on sequence-specific hydrogen bonds. In many cases, only limited experimental data for the interface interaction are available, so it was necessary to study the effect of fewer AIRs for docking. Therefore, for the third AIR set, we aimed to find the minimum AIRs needed for successful docking. We first used one AIR derived from an N-terminal residue of α-helix and its interacting base in each domain for docking calculation because the N-terminal α-helix is known to fit into the major groove of the DNA in the Zif268-DNA complex [27]. However, use of one AIR in each domain can generate only a few wrap-around models. Apparently, one AIR in each domain is not enough to cover the interface of the complex. To represent the entire surface of each α-helix in the interface, we thus used two AIRs in each domain, one in the N-terminus and the other in or near the C-terminus of the α-helix. The detailed selection of the two AIRs in each domain to generate an efficient zinc-finger protein complex model is described in the section Docking Procedure.

After the three different AIR sets were derived, the docking calculations were performed, and the generated complex models were analyzed in terms of wrap-around conformation, localization of AIRs in true and false complex models, and energy of AIR (E_AIR_) distribution. Finally, the top 10 structures were selected on the basis of HADDOCK score and analyzed on the basis of iRMSD, E_inter_, BSA and F_nat_. The same docking procedures were used for other test cases, such as YY1, WT1 and Aart, to confirm whether this approach can be used to model other zinc-finger-DNA complexes. Furthermore, the same approach was used to model the previously unreported Sp1-DNA complex.

### Wrap-around conformation of the complex models for different AIR sets

Wrap-around conformation is the unique DNA binding mode for the three-Cys_2_His_2 _zinc-finger protein. Thus, we checked whether the modeled Zif268-DNA complex forms a wrap-around conformation using the Pymol program. For each AIR set, we analyzed the number of wrap-around conformations in 200 structures (Table 1). For the first AIR set, only 50 of 200 complex models showed wrap-around conformation, the lowest among all three AIR sets. The remaining 150 complex models were considered false models. For the second AIR set, only 56 of the 200 structures showed wrap-around orientation. For the third AIR set, the number of wrap-around models was greatly increased (100% of the models). Together, these results indicate that three different AIR sets can all generate wrap-around orientation models, and the third AIR set generates a significantly high number of wrap-around models. Thus, the third AIR set, that is, two AIRs in each domain, is a better AIR set because of the number of wrap-around conformations obtained.

**Table 1:** The 10 best Zif268-DNA complex models for each AIR set were selected on the basis of HADDOCK score. Standard deviations are shown as subscripts.

AIR Set	Wrap-around^a ^conformation	iRMSD^b ^(Å)	iRMSD^c ^(Å)	HADDOCK score^d^	E_inter_^e ^(kcal mol^-1^)	BSA^f^ (Å^2^)	F_nat_^g^
(i)	50/200	2.22_0.34_	7.19_3.84_	-241.91_6.39_	-1012.96_44.52_	2797.64_72.76_	0.71_0.05_

(ii)	56/200	2.62_0.27_	6.84_2.59_	-189.02_13.76_	-820.31_56.69_	2428.23_98.19_	0.58_0.03_

(iii)	200/200	2.14_0.27_	2.28_0.20_	-238.62_4.54_	-999.27_35.34_	2780.30_103.50_	0.72_0.04_

^a^Number of wrap-around models from analysis of 200 complex models.

^b^interface Root Mean Square Deviation for the 10 best models.

^c^interface Root Mean Square Deviation for the 200 models.

^d^HADDOCK score was calculated as a weighted sum of intermolecular electrostatic, van der Waals contacts, desolvation, AIR energies and a buried surface area term.

^e^Intermolecular energy.

^f^Buried surface area.

^g^Fraction of native contacts.

### Localization of AIRs in the complex models and geometric distribution of AIR sets in the reference structure

**Figure 1:**
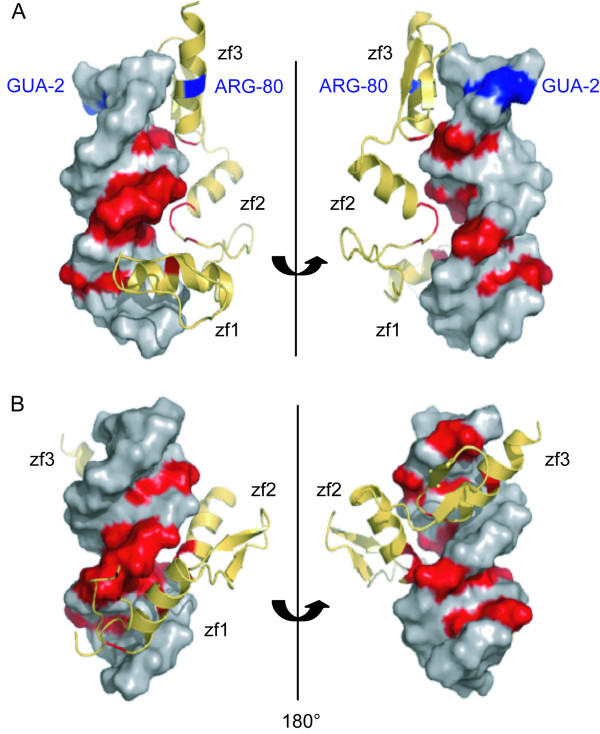
**Example of false and true complex models generated based on the second ambiguous interaction restraint (AIR) set**. Red and blue indicate the matching and mismatching AIRs between residues and bases, respectively. (A) False complex model: the AIR for Arg80 and GUA2 is mismatched, which results in a complex model in which the protein does not wrap around DNA. (B) True complex model: all the AIRs between residues and bases are nearly matched.

**Figure 2:**
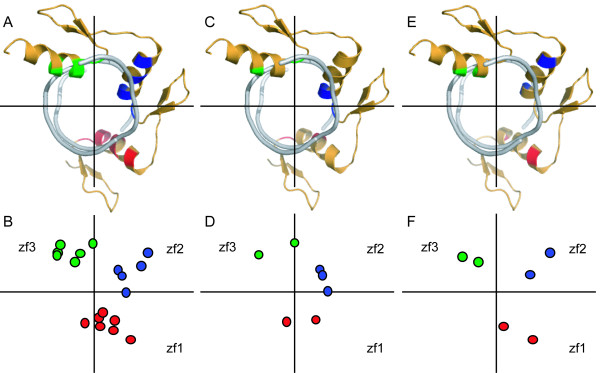
**The distribution of the AIR sets used for docking the Zif268-DNA complex is shown as the top and projection views**. In the top view, down the DNA helix axis, the AIRs in each zinc finger domain are marked in different colors: finger 1 (red), finger 2 (blue) and finger 3 (green). To better view the AIR distribution in each domain, two lines, which intersect each other in the DNA helical axis that separates each domain, were drawn. In the projection view, each AIR in the domain is represented as a dot. (A) Top view of the first AIR set in the complex, (B) projection view of the first set of AIRs, (C) top view of the second AIR set in the complex, (D) projection view of the second set of AIRs, (E) top view of the third AIR set in the complex, and (F) projection view of the third set of AIRs.

We analyzed the association of localization of AIRs in the false complex models and geometric distribution of AIRs in the crystal complex structure. Analysis of the false models from use of the first and second AIR sets revealed some localization of AIRs mismatched between protein and DNA. Examples of localization analysis in the false and true models generated by the second AIR set are shown in Figure 1A and 1B, respectively. In the true models, all the spatial localizations of AIR-related residues and bases nearly matched, whereas in the false models, the spatial localization of the AIR between Arg80 of finger 3 and GUA2 did not match, despite the localizations of the remaining AIR-related residues and bases being relatively matched. Because of this single mismatch, the protein is unable to wrap around the DNA. To explore the association of localization of AIR-related residues and bases in complex models and geometric distribution of AIRs in the complex structure, we analyzed the geometric distribution of AIRs in different sets (the description of geometric distribution analysis is in the Methods section). For the first AIR set, the top view in Figure 2A shows the distribution of residues for the AIRs in each domain with reference to the DNA helix axis. In the simplified projection view in Figure 2B, each dot represents the residue in the AIRs in the corresponding domain. The number of AIRs in each zinc-finger domain varies: 7 AIRs in the first zinc finger, 5 in the second, and 6 in the third. Altogether, 18 AIRs were used to represent the complete interface of the complex; however, the geometric distribution of the AIRs among the three domains is not equal in space. This imbalance creates a bias in the interface between each domain and DNA, which ultimately affects the spatial orientation of the protein-DNA complex and results in a reduced number of wrap-around conformations. The distribution of AIRs in each domain of the second AIR set is shown in Figure 2C and 2D. Although the total number of AIRs is less than that in the first set, the geometric distribution in space is still unequal among the domains and leads to approximately 75% false models. The example of the false complex model based on this set showed a spatial localization of the AIR mismatched between Arg80 of finger 3 and GUA2 (Figure 1). We also found that the AIR is out of the major cluster in unequal geometric distribution. Only AIRs that form a cluster in a local geometric region lead to a match in rigid body docking. The geometric distribution of the AIRs in the interface for the third AIR set is shown in Figure 2E and 2F and reveals that the AIRs among the domains are relatively equal, with no false model found for this AIR set. Therefore, the number of AIRs in each domain has a direct effect on the geometric distribution of AIRs among domains. For unequal distribution of AIRs, only AIRs that form a cluster in a local geometric region lead to a match in rigid body docking. The unequal number of AIRs in each domain affects the overall AIR distribution and results in mismatching during docking. Our data support that the relative equivalent distribution of the AIRs among the domains is essential to increase the number of wrap-around conformations. Thus, the refinement of AIRs in terms of number and position among the domains is important to increase the unique fraction of docking model for the classical three-Cys_2_His_2 _zinc-finger protein that binds DNA in a wrap-around conformation.

### Analysis of complex models based on AIR energy

Our main focus in this work was to assess the effect of various AIR sets in obtaining good complex models. Although the geometric distribution analysis provided valuable information for the different AIR sets, it could not give a complete understanding of whether the derived AIRs are matched or not in the complex models. Instead, E_AIR _analysis of the complex models is more precise and shows the suitability of the AIRs for docking. In brief, if the distance between the AIRs is large, the E_AIR _value is high and indicates that the AIRs do not satisfy the distance criteria that lead to mismatched AIRs, as well as a non-wrap-around complex model. So the E_AIR _in each complex model is a good indicator of the suitability of AIR sets for generating a complex model. To understand the E_AIR _distribution in the final 200 complex models assessed, we produced a plot of the HADDOCK score as a function of E_AIR_.

**Figure 3:**
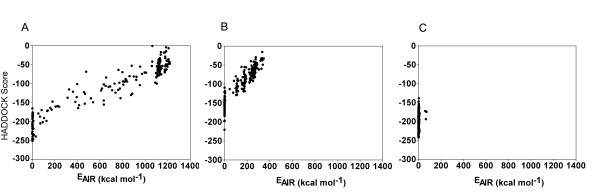
**HADDOCK score versus AIR energy (E_AIR_) plot for the Zif268-DNA complex model based on (A) the first AIR set, (B) the second AIR set, and (C) the third AIR set**. The filled circle corresponds to the individual structure. The HADDOCK score corresponds to the weighted sum of intermolecular electrostatic, van der Waals contacts, desolvation, E_AIR_, and a buried surface area term.

The plots (Figure 3) display the unique fraction solution in each case. With the first AIR set (Figure 3A), two populations are revealed, one with low E_AIR _and the other with high E_AIR_, although the distribution is broad. Structures in the high-E_AIR _population contained many mismatched AIRs, and the low-E_AIR _population contained fewer structures but with no AIR mismatches. With the second AIR set, in general, four populations were obtained (Figure 3B), with the best population possessing the lowest E_AIR_. By contrast, only one unique fraction of the complex structures (Figure 3C) with low E_AIR _was observed with the third AIR set. Analysis of this population revealed no mismatches between residues and bases. Thus, complex models generated on the basis of two AIRs in each domain showed a major population with low E_AIR _value, which indicates that use of two AIRs in each domain for docking calculation is more suitable than use of other AIR sets.

### Comparison of the 10 best complex models to the reference structure

**Figure 4:**
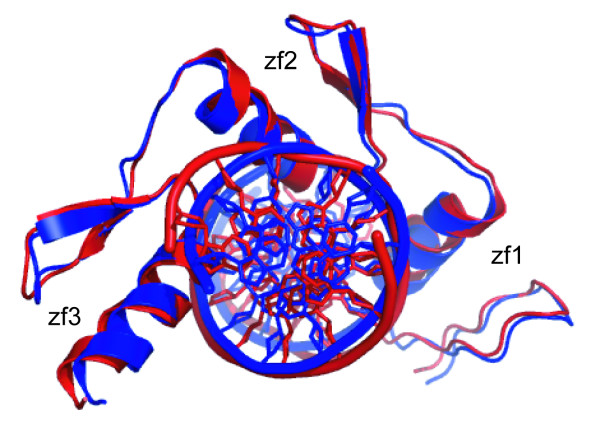
**The best docking Zif268-DNA complex model (blue) generated on the basis of the third AIR set superimposed on the reference structure (red) on all heavy atoms**. The RMSD of the entire complex is 1.29 Å. The protein and DNA bases are shown in cartoon and cartoon-ring mode, respectively, by the Pymol program.

The 10 best complex models for each AIR set were selected on the basis of HADDOCK score. The mean iRMSD, E_inter_, BSA and F_nat _values for all 10 structures are in Table 1. The mean iRMSD for the 10 best complex models based on the first and third AIR sets was 2.22 and 2.14 Å, respectively. We also calculated the mean iRMSD for all 200 structures for each AIR set and found that the value based on the third AIR set (2.28 Å) was better than that based on the other two sets. The E_inter _values for the first and third sets are compatible and are better than those for the second set. The BSA values for the first and third sets are similar to that for the reference structure (2645.49 Å^2^). The F_nat _for the third AIR set is similar to the first AIR set. Overall, the first and third AIR sets are better able to generate complex models evaluated by iRMSD, BSA and F_nat _with respect to the reference structure. The best Zif268-DNA complex model based on the third AIR set was superimposed on the reference structure (Figure 4). Use of the second type of AIR set was not able to achieve significant improvement in terms of wrap-around number, iRMSD, BSA or F_nat _as compared with the other AIR sets. Although the 10 best complex models with the first and third AIR sets are similar, the wrap-around conformation (true model) largely occurred with the third AIR set (100%), as compared with the models for the first AIR set (25%). Therefore, the convergence of the docking model with the third set is much better than with the first set. Even if complete interface information is used to formulate AIRs for docking, the number of wrap-around conformations is significantly reduced in the final 200 structures. The two AIRs for each domain, with a reasonable geometric distribution of the AIRs, are sufficient to generate wrap-around complex models.

### Complex modeling of other test cases, YY1, WT1 and Aart

We also extended this method to analyze other classical Cys_2_His_2 _zinc-finger proteins with known crystal structures, YY1 (PDB code: 1UBD), WT1 (PDB code: 2JP9) and Aart (PDB code: 2I13). For these cases, we used only three zinc fingers important for DNA sequence specific binding in complex modeling with a canonical B-DNA. The docking was performed with the two AIRs in each domain. The procedure for selecting the two AIRs in each domain is described in the following section. The results for these test cases are in Table 2 and show similar results to that for the Zif268-DNA complex models, thus further confirming that two AIR restraints in each domain are sufficient to generate good complex models.

**Table 2:** Data for the 10 best complex models for other test cases, such as YY1, WT1 and Aart, generated with two AIRs in each domain. Standard deviations are shown as subscripts.

Complex names	Wrap-around^a ^conformation	iRMSD^b ^(Å)	iRMSD^c ^(Å)	HADDOCK score^d^	E_inter_^e ^(kcal mol^-1^)	BSA^f^ (Å^2^)	F_nat_^g^
YY1	173/200	1.77_0.26_	2.07_0.36_	-154.34_2.52_	-110.77_35.68_	2659.42_58.95_	0.76_0.04_

WT1	189/200	2.06_0.24_	2.14_0.22_	-213.34_6.69_	-749.97_60.01_	2757.10_56.41_	0.76_0.04_

Aart	200/200	2.74_0.19_	2.76_0.21_	-222.67_4.04_	-819.68_54.57_	2896.50_121.53_	0.73_0.05_

^a^Number of wrap-around models from analysis of 200 complex models.

^b^interface Root Mean Square Deviation calculated for the 10 best models.

^c^interface Root Mean Square Deviation calculated for the 200 models.

^d^HADDOCK score was calculated as a weighted sum of intermolecular electrostatic, van der Waals contacts, desolvation, AIR energies and a buried surface area term.

^e^Intermolecular energy.

^f^Buried surface area.

^g^Fraction of native contacts.

### Complex modeling based on the homology modeled structure

The above-mentioned complex models were all generated on the basis of structures of the bound zinc finger proteins derived from known crystal complex structures. One may wonder if the approach is also applied when the free form structure or the homology structure is used as the starting structure. It is therefore worthwhile to check them. However, the linker regions of the free Cys_2_His_2 _zinc finger proteins are highly flexible so that 3 D structure of the free form structure of Zif268 as well as other three-Cys_2_His_2 _zinc finger proteins is not available. We therefore used the homology modeled structure as an initial structure to perform docking calculation. Since the structural alignment of the bound Zif268 protein with other bound zinc-finger proteins has RMSDs of 1.413 Å, 0.745 Å, and 0.992 Å for YY1, Aart, and WT1, respectively, and the sequence identities among these proteins are varied, in the range of 63% (Zif268-WT1) to ~ 41% (Zif268-YY1). To obtain a detailed analysis, three homology model structures for each protein were generated. For example, three homology modeled structures of Zif268 were generated using the bound-WT1, AART and YY1 structure as an individual template, respectively. In total, 12 homology modeled structures were made. For each case, the AIRs were obtained by using the procedure described in the following paragraph and then docking was performed. The 10 best complex models in each case were analyzed and the results are shown in Additional file 1-Table S1. The iRMSD and F_nat _for the 10 best complex modes in each case are within the range of 1.86-2.86 Å and 0.54-0.77. These results are acceptable and comparable to those based on the bound form docking, demonstrating that the homology modeled structure can also be applied as a starting structure to generate a three-Cys2His2 zinc finger-DNA complex model using our approach.

### An efficient docking procedure to generate a zinc-finger protein-DNA complex model

**Figure 5:**
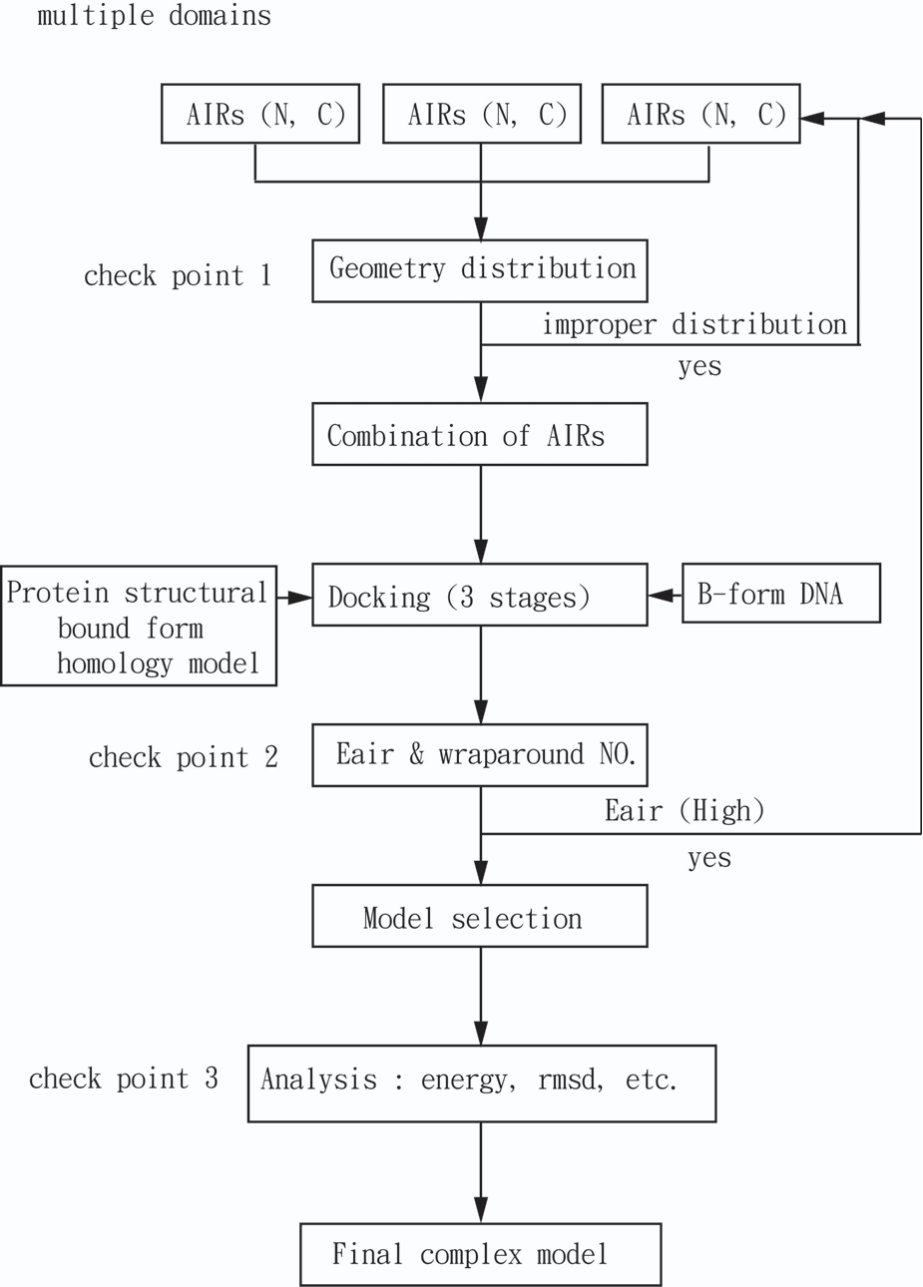
**Flowchart of the stepwise procedure for modeling the three-Cys_2_His_2 _zinc-finger-DNA complex**.

From the complex modeling of Zif268 and the other test cases YY1, WT1 and Aart, we derived a stepwise procedure to generate a complex model for the three-Cys_2_His_2 _zinc-finger proteins (Figure 5).

The first step, which is the most important in generating a complex model, is the selection of two AIRs in each domain. Two AIRs, one in the N-terminus and another in or near the C-terminus of the α-helix in each domain, should be selected on the basis of the available experimental data or bioinformatics prediction. Of note, only a few residues in the N and C-termini of the α-helix in each domain interact with DNA. If the user has this complete information, then the selection of AIRs has few combinations. Each AIR set can give a different result, so identifying the suitable AIR set that can generate a complex model is necessary. The following steps are used to identify the suitable AIRs to generate a complex model.

The second step is the analysis of the geometric distribution of the AIRs. From modeling the Zif268-DNA complex and other test cases, we found that two AIRs in each domain with a reasonable geometric distribution can generate a complex model. So the geometric distribution analysis is a prescreening procedure to filter the few combinations of AIRs with improper distribution. The improper distribution is mainly caused by some AIRs located in only one side of the DNA. The projection view of the AIRs is used to analyze this distribution. For analysis of the unknown case that does not have a complex structure, a homology-modeled protein structure is necessary. The homology-modeled structure can be superimposed on its published homologous structure. This superimposition can reveal the DNA axis, which can be used as a reference to analyze the AIR distribution. However, a few AIR sets can show similar spatial orientation in the projection view. Thus, the only way to identify the best AIR set is by calculating docking with all these sets individually. Each AIR set can give different results, because the AIR is an atom-to-atom restraint; analyzing this information by only the projection view is difficult, so the following step is necessary to identify the best AIR set.

The third step is the analysis of the wrap-around conformation and E_AIR_. This analysis will help determine the suitability of the AIRs for generating a complex model. Each AIR set can give different numbers of wrap-around conformation models. Among the AIR sets, the one that can generate more wrap-around conformations and the occurrence of a single major population of complex models with low AIRs energy in the E_AIR _analysis reveals the AIR set that is the best for generating the complex model. In case of few numbers of wrap-around models and only a few models in the population with low E_AIR _values, the user should go back to the first step to choose another AIR pair for docking.

The final step is the analysis of the 10 best complex models. After successful docking, the 10 best complex models are selected on the basis of the HADDOCK score, and these models are analyzed for iRMSD and F_nat _with respect to the reference structure only if the reference structure is available. For the unknown cases that do not have a complex structure, analysis of E_inter_, RMSD (from lowest energy minimum models) and qualitative comparison with other experimental data can help to validate the model.

Our study revealed that two AIRs in each domain is the minimum information required to efficiently generate a good complex model; however, to identify the best AIRs that can provide a complex model, a few rounds of docking are needed. We used these procedures to model the previously unreported Sp1-DNA complex.

### Analysis of Sp1-DNA complex model

**Figure 6:**
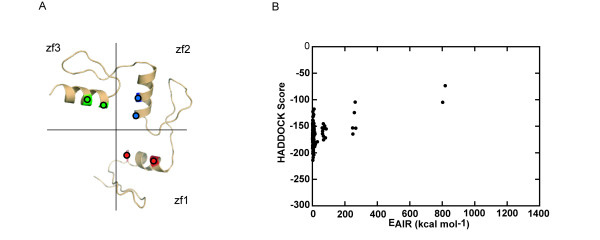
**AIR geometric distribution and E_AIR _analysis of Sp1-DNA complex models. ** (A) The distribution of the AIR set used for docking of the Sp1-DNA complex is shown as the projection view. The AIRs in each zinc finger domain are marked in different colors: finger 1 (red), finger 2 (blue) and finger 3 (green). (B) HADDOCK score versus E_AIR _for the Sp1-DNA complex models.

**Figure 7:**
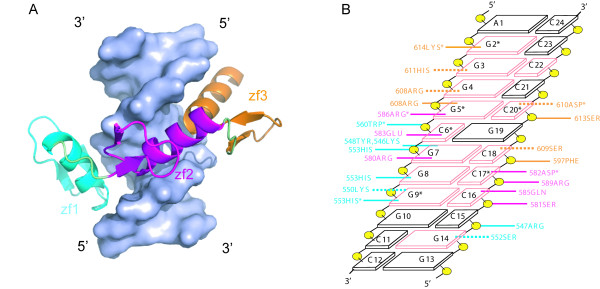
**Best Sp1-DNA complex model and detailed interaction scheme**. (A) The best Sp1-DNA complex model generated on the basis of two AIRs in each domain. (B) The interacting residues in zinc fingers 1, 2 and 3 are cyan, magenta and orange, respectively. The interacting DNA bases are salmon. The solid lines and dotted lines indicate the hydrogen bonds and non-bond contacts, respectively. The residues and bases used as AIRs for docking are marked with an asterisk.

The Sp1-DNA interaction has been extensively studied by various experimental methods. For example, the hydrogen bonds and non-bond contacts between Sp1 and DNA were reported by structural comparison with the Zif268-DNA complex [24], and these reported interactions (Additional file 1-Figure S1) are consistent with those from ethylation interference analysis [25], methylation interference analysis and mutation study [26]. However, until now, the complex model for this system by docking has not been reported. In this study, we used the reported interactions to derive AIRs with a reasonable geometric distribution for docking (Figure 6A). Analysis of the final complex models revealed that 193 of 200 structures were in wrap-around conformation. The analysis based on E_AIR _(Figure 6B) showed most of these structures are present in a single population. The 10 best complex models were chosen on the basis of the HADDOCK score. Figure 7A shows the best model for the Sp1-DNA complexes (Additional file 2), whereby the α-helix of each zinc finger fits directly into the major groove of the DNA. Except for finger 1, fingers 2 and 3 have identical residues at positions -1 and 2 (Arg and Asp) as compared with those for Zif268, and these residues make coordinated DNA base contacts. Figure 7B shows the detailed interactions for at least 5 of the 10 best complex models.

For finger 2, residues Arg580, Gly583 and Arg586 form hydrogen bonds with bases GUA7, CYT6 and GUA5, respectively, in the primary strand of the DNA, whereas Asp582 contacts CYT17, Gln585 contacts CYT16 and Ser581 contacts the sugar phosphate backbone of CYT16 in the complementary strand. These observations are consistent with the reported interactions [24] (Additional file 1-Figure S1). However, ethylation interference analysis [25] revealed that Arg565 interacts with the phosphate between GUA3 and GUA4, but we did not observe this interaction in our model. For finger 3, residues Arg608 and Lys614 form hydrogen bonds with GUA5 and GUA2, respectively. His611 and Asp610 form only nonbond contacts with bases GUA3 and CYT20, whereas in the reported interaction [24] (Additional file 1-Figure S1), these two form hydrogen bonds with GUA3 and CYT20. Ethylation interference analysis [25] revealed that Lys595 interacts with the phosphate between GUA9 and GUA10. However, we did not observe this interaction in our model. As compared with the reported interactions [24], one new interaction was observed between Phe597 and CYT18 in our complex model. For finger 1, residues Lys550 and His553 form hydrogen bonds with two bases each, GUA9 and GUA10, and GUA8 and GUA9, respectively, in the reported interactions [24] (Additional file 1-Figure S1). However, in our model, we observed only the His553 interaction, and Lys550 formed only a nonbond contact with GUA9. Our model is consistent with that from methylation interference analysis [26] suggesting that Lys550 interacts with GUA9/10. Apart from this finding, all other backbone contacts are consistent with reported interactions. Overall, our complex model is almost consistent with the reported model interactions, so the model generated by our approach is acceptable. As well, much less information was used to generate this complex model.

Practically, obtaining such precise pairwise information seems difficult, so in our study, we also included the non-pairwise AIRs to model the Sp1-DNA complex (Table 3). This analysis showed a decrease in number of wrap-around conformations with non-pairwise AIR sets as compared with pairwise AIR sets, which suggests that the pairwise AIR set is better than the non-pairwise set in calculating docking. Accordingly, if the pairwise set is not available for docking calculation, then the non-pairwise set could be used to generate a complex model but may not obtain as good a result as that generated by use of the pairwise set.

**Table 3:** Data for the 10 best structures of the Sp1-DNA complex models generated with pairwise and non-pairwise AIR sets. Standard deviations are shown as subscript.

AIR types	Wrap-around^a ^conformation	RMSD^b ^(Å)	HADDOCK score^c^	E_inter_^d ^(kcal mol^-1^)	BSA^e^ (Å^2^)
Pairwise	193/200	1.22_0.47_	-200.55_6.52_	-858.33_32.43_	2165.77_102.56_

Non-pairwise	167/200	1.26_0.80_	-206.07_4.54_	-846.01_42.94_	2273.60_125.63_

^a^Number of wrap-around models from analysis of 200 complex models.

^b^Root Mean Square Deviation calculated for the 10 best models from the lowest energy structure.

^c^HADDOCK score was calculated as a weighted sum of intermolecular electrostatic, van der Waals contacts, desolvation, AIR energies and a buried surface area term.

^d^Intermolecular energy.

^e^Buried surface area.

## Conclusions

Formulating optimal AIRs in each domain to successfully model a three-Cys_2_His_2 _zinc-finger-DNA complex by use of HADDOCK requires only a limited amount of interaction information. Although all restraints in the three different AIR sets were derived on the basis of the real interactions observed in the crystal structure, the quality of docking results varies. The results for different AIR sets showed that the unequal distribution in one domain largely affects the other two domains in three-Cys_2_His_2 _zinc-finger domains during docking. Therefore, balancing the AIRs in each domain is necessary, as is the overall interface. Analysis of the geometric distribution of AIRs, wrap-around conformation, E_AIR _versus HADDOCK score, iRMSD, and F_nat _revealed that two AIRs for each domain, with a reasonable geometric distribution, is sufficient to successfully generate a complex model. By comparison to the reference structure, we are confident that the complex model of Zif268-DNA, as well as those for other test cases, generated with HADDOCK is acceptable and reliable. We also generated the Sp1-DNA complex model for the first time using this approach. Most of the interactions in this model are consistent with the reported interactions. The approach we describe to model the three-Cys_2_His_2 _zinc-finger Sp1-DNA can be easily applied to model other similar three-Cys_2_His_2 _zinc-finger proteins with complex structures unknown to date.

Zinc-finger proteins are the largest family of nucleic acid binding proteins in eukaryotes [28], but only a small number of the three-Cys_2_His_2 _zinc-finger protein-DNA complex structures have been studied. Because obtaining all the interface contacts from experiments is tedious and difficult, using fewer AIRs with a reasonable geometric distribution to generate zinc-finger protein-DNA complex models in which the protein wraps around DNA is greatly beneficial and can facilitate computational studies to better understand the zinc-finger protein-DNA interactions. As well, this approach further demonstrates the versatility of using HADDOCK for computational modeling.

## Methods

### Starting structure of Zif268, Sp1 protein and DNA

The coordinate file of the Zif268-DNA complex was obtained from the RCSB Protein Data Bank [29] (PDB code: 1ZAA), and the coordinate of the bound Zif268 was separated and used as the starting structure. The DNA in this complex has overhanging bases (Additional file 1-Figure S2), and during canonical B-DNA construction, it was converted to paired bases by including the complementary bases by use of the nucleic acid modeling module in Discovery studio 2.0 (Accelrys). Similarly, the consensus DNA sequence of Sp1 binding (5'-AGGGGCGGGGCC-3') was built. The two constructed DNAs were assigned as a single chain identifier and renumbered. Atom and residue names were matched to the topallhdg5.3.pro [30] and dna-rna_allatom.top topology file naming for direct use in HADDOCK. The homology model of Sp1 was constructed by use of the Modeller module in Discovery studio 2.0 (Accelrys). The structures from PDB (1alf, 1mey and 1jk1) [31] were chosen as templates for modeling.

### AIRs for docking Zif268, YY1, WT1, Aart and Sp1

The AIRs derived from any kind of experimental data or bioinformatics prediction can provide information about the interacting residues in the interface of the complex. The residues of AIRs can be defined as active or passive. Active residues are identified from experiments or bioinformatics analysis, and passive residues are surface neighbors of the active residues. An AIR is defined as an ambiguous intermolecular distance (diAB) with a maximum value of, typically, 2 Å between any atom "m" of an active residue "i" of component A (miA) and any atom "n" of both active and passive residues "k" (Nres in total) of component B (nkB) (and inversely for component B) [9]. The effective distance *d*_iAB_^eff ^for each restraint is calculated with the following equation:

where *N*_atoms _indicates all atoms of a given residue and *N*_res _is the sum of active and passive residues for a given molecule. The AIRs are incorporated as an additional energy term in the HADDOCK score. If the residues and bases for each AIR are far away, then the effective distance for each restraint increases and the E_AIR _is also increased. For DNA binding proteins possessing multiple domains, the overall E_AIR _will be greatly affected, even if a single AIR is unable to satisfy the distance criteria.

In general, the AIR setup is created with all possible combinations of active and passive residues. This setup allows the HADDOCK program to search all the possible configurations around the defined residues. However this default AIR setup may not be suitable for proteins with multiple domains. For example, for the three-Cys_2_His_2 _zinc finger, the use of AIRs allows for the residues of zf1 to combine with DNA bases that interact with zinc fingers 2 and 3. The same kinds of combinations are generated for zinc finger 2 and 3 domains. Obviously, these kinds of combinations may not allow the protein to find suitable configurations in the interface region, which results in a protein that may not wrap around DNA. So in our approach, we defined the AIRs for local regions for each zinc-finger domain and its corresponding interacting region in DNA. Then we summed all the AIRs in the three domains as a single input for docking.

For Zif268-DNA docking, we used three different sets of AIRs, as shown in Table 4. Information on hydrogen bonds and van der Waals contacts in the interface between Zif268 and DNA (Additional file 1-Table S2) and for YY1, WT1 and Aart were analyzed on the basis of the crystal structure from HBPLUS [32]. The AIR table for the test cases YY1, WT1, and Aart is in Additional file 1-Table S3. For the unknown complex structure of Sp1, we used the reported interaction information [24] to select the active residues for AIRs (Additional file 1-Table S4). In this study, we defined the AIRs in a pairwise manner for docking Zif268 and for other test cases. For many cases, obtaining such explicit knowledge about the specific pairwise interaction may not be easy. To demonstrate this, we also used nonpairwise AIR sets for analyzing the docking of the Sp1-DNA complex.

**Table 4:** Three AIR sets used for docking Zif268 with DNA

No	Molecule	Zinc finger 1	Zinc finger 2	Zinc finger 3
(i)	Zif268	R18, S19, D20, E21, R24, I28, H25	R46, D48, H49, H53, T56	H74, S75, D76, E77, K79, R80
	DNA	G10, T13, T13, G8, G8, G6, G7	G7, C17, G6, G4, C3	G4, C18, A20, G2, C19, G2

(ii)	Zif268	R18, R24	R46, D48, H49	R74, R80
	DNA	G10, G8	G7, C17, G6	G4, G2

(iii)	Zif268	S19, I28	D48, T56	D76, R80
	DNA	T13, G6	C17, C3	A20, G2

(i) The first AIR set was derived on the basis of hydrogen bonds and van der Waals contacts in all residues and bases from the interface of Zif268-DNA X-ray crystal structure. (ii) The second AIR set was derived on the basis of sequence-specific hydrogen bonds between the residues and bases. (iii) Two AIRs for each domain in the third AIR set were derived on the basis of van der Waals or hydrogen bond contacts.

### Geometric distribution analysis of different sets of AIRs

To simplify the analysis of the geometric distribution of the three AIR sets in Zif268-DNA, the following considerations were applied. As the protein wraps around the DNA along the major groove, the DNA helix axis was considered the reference axis for the geometric distribution of AIRs. Because the residues and bases in AIRs are extremely close in proximity, for clarity, we considered only the geometric distribution of the residues in the AIRs with reference to the DNA helix axis. The geometric distribution of the AIRs in 3-D space is difficult to represent, so we simplified this into a 2-D representation with reference to the DNA helix axis without losing distribution information of the AIRs. For the unknown complex of Sp1, we superimposed the homology protein structure on the Zif268-DNA crystal structure and then obtained the DNA helix axis and used that axis as a reference for geometric distribution analysis of AIRs in Sp1.

### Docking procedure

The docking procedure consisted of three stages: rigid-body docking, semi-flexible refinement and final refinement in explicit solvent. During the rigid-body docking, 1000 complex models were generated for each set of AIRs. The best 20% complex models were selected on the basis of HADDOCK score defined as a weighted sum of intermolecular electrostatic, van der Waals contacts, desolvation, E_AIR _and BSA term [33]. These models were used for further refinement in the semi-flexible refinement stage consisting of three parts: rigid-body torsion angle dynamics, semi-flexible simulated annealing stage and final semi-flexible simulated annealing stage. The final stage of the docking protocol is gentle water refinement. The effects of global and local flexibility of the DNA during docking have been reported [10]; thus, the default option was used to define the flexible regions of DNA. Also, default HADDOCK parameters were used, except for the random deletion of a fraction of the restraint option, which was set as false for all docking calculations. Additional restraints to maintain base planarity and Watson-Crick bonds were introduced for the DNA.

### Analysis of the complex models

For each docking, the wrap-around orientation of the complex models was analyzed by use of the Pymol program [34]. The final 200 structures were analyzed according to E_AIR _versus HADDOCK score. The 10 best complex models were then selected on the basis of HADDOCK score. The iRMSD values of the complex interface were calculated by the McLachlan algorithm [35] as implemented in the Profit program (Martin, A.C.R., http://www.bioinf.org.uk/software/profit/). All heavy atoms were used to calculate the iRMSD of the complex interface. Intermolecular contacts were evaluated with a 5 Å cut-off value [10]. The F_nat _was defined as the number of native intermolecular contacts on a nucleotide-residue basis (hydrogen bonded and non-bonded) identified in a docking solution, divided by the total number of contacts in the reference structure. Both BSA and E_inter _were analyzed for the 10 best complex models for each AIR set.

## Authors' contributions

CCC and CC constructed the idea of three-Cys_2_His_2 _zinc-finger-DNA docking. CCC and MR performed the docking and the following analysis. All authors participated in drafting the manuscript and approved the final version.

## Supplementary Material

Additional file 1 **Supplementary Tables and Figures**. This file contains 4 Tables and 2 Figures. Table S1 lists the statistics analysis for the 10 best complex models which were generated based on homology modeled structure. Table S2 (A) and (B) lists the intermolecular hydrogen bonds and van der Waals contacts of Zif268-DNA crystal structure. Table S3 lists the AIR set used for docking of YY1, WT1 and Aart with their interacting DNAs. Table S4 lists the AIR sets used for docking of Sp1 with DNA. Figure S1 displays the reported binding mode of Sp1-DNA complex. Figure S2 (A) and (B) displays the sequence of Zif268 and its interacting DNA. Click here for file

Additional file 2 **Structural coordinates of Sp1-DNA complex models**. This file contains the structural coordinates of the best 10 Sp1-DNA complex models in PDB format. Click here for file
