# Supplementary material for: An effective approach for generating a three-Cys2His2 zinc-finger-DNA complex model by docking
Source: BMC Bioinformatics. 2010 Jun 18;11:334. doi: 10.1186/1471-2105-11-334 (PMC2905368; doi:10.1186/1471-2105-11-334)
Supplement: Additional file 1 — Supplementary Tables and Figures. This file contains 4 Tables and 2 Figures. Table S1 lists the statistics analysis for the 10 best complex models which were generated based on homology modeled structure. Table S2 (A) and (B) lists the intermolecular hydrogen bonds and van der Waals contacts of Zif268-DNA crystal structure. Table S3 lists the AIR set used for docking of YY1, WT1 and Aart with their interacting DNAs. Table S4 lists the AIR sets used for docking of Sp1 with DNA. Figure S1 displays the reported binding mode of Sp1-DNA complex. Figure S2 (A) and (B) displays the sequence of Zif268 and its interacting DNA. [file 1471-2105-11-334-S1.DOC]

**Supplementary Data**

**Table S1 –** Statistics analysis for the 10 best complex models for each test case which were generated based on homology modeled structure. Standard deviations are shown as subscripts.

| Initial homology model | Template | Wraparound^a^ conformation | iRMSD (Å)^b^ | iRMSD (Å)^c^ | HADDOCK^d^ score | E_inter_^e^ (kcal mol^–1^) | BSA(Å^2^)^f^ | F_nat_^g^ |
| --- | --- | --- | --- | --- | --- | --- | --- | --- |
| Zif268 | WT1 | 188/200 | 2.32_0.17_ | 2.61_0.46_ | -213.20_4.83_ | -835.38_41.38_ | 2773.99_115.12_ | 0.64_0.04_ |
| Zif268 | AART | 157/200 | 2.35_0.26_ | 2.82_0.65_ | -210.89_11.06_ | -870.01_55.54_ | 2762.49_121.68_ | 0.61_0.04_ |
| Zif268 | YY1 | 170/200 | 2.51_0.20_ | 2.80_0.88_ | -209.97_5.16_ | -894.91_24.44_ | 2625.24_90.54_ | 0.58_0.05_ |
| YY1 | AART | 175/200 | 2.29_0.35_ | 2.66_0.95_ | -138.67_10.79_ | -64.55_76.66_ | 2599.11_111.96_ | 0.60_0.10_ |
| YY1 | WT1 | 189/200 | 1.86_0.19_ | 1.90_0.18_ | -162.88_2.83_ | -217.05_47.43_ | 2741.21_90.02_ | 0.73_0.04_ |
| YY1 | Zif268 | 187/200 | 2.43_0.21_ | 2.50_0.38_ | -141.73_2.89_ | -43.10_36.31_ | 2695.61_103.77_ | 0.61_0.03_ |
| WT1 | Zif268 | 199/200 | 2.12_0.13_ | 2.39_0.34_ | -216.52_5.89_ | -1021.44_50.83_ | 2763.46_81.33_ | 0.77_0.04_ |
| WT1 | AART | 151/200 | 2.83_0.16_ | 3.21_0.37_ | -162.53_4.79_ | -564.10_37.92_ | 2439.34_83.64_ | 0.54_0.04_ |
| WT1 | YY1 | 185/200 | 2.60_0.17_ | 2.95_0.56_ | -177.23_7.38_ | -969.13_46.31_ | 2327.99_98.95_ | 0.58_0.06_ |
| AART | Zif268 | 128/200 | 2.86_0.15_ | 3.30_0.91_ | -217.09_0.10_ | -875.10_44.21_ | 2770.33_68.98_ | 0.73_0.22_ |
| AART | WT1 | 140/200 | 2.81_0.32_ | 3.65_0.56_ | -184.58_11.90_ | -694.31_53.91_ | 2521.87_115.08_ | 0.59_0.65_ |
| AART | YY1 | 150/200 | 2.47_0.13_ | 2.69_0.66_ | -203.68_6.00_ | -772.15_30.06_ | 2658.65_110.73_ | 0.70_0.09_ |

^a^Number of wrap-around models from analysis of 200 complex models.

^b^interface Root Mean Square Deviation for the 10 best models.

^c^interface Root Mean Square Deviation for the 200 models.

^d^HADDOCK score was calculated as a weighted sum of intermolecular electrostatic, van der Waals contacts, desolvation, AIR energies and a buried surface area term.

^e^Intermolecular energy.

^f^Buried surface area.

^g^Fraction of native contacts.

**Calculation of hydrogen bonds and van der Waals contacts in the complex structure**

Information on hydrogen bonds and van der Waals contacts in the interface of the Zif268–DNA complex is required to derive the different sets of AIRs. The HBPLUS [32] program was used for analysis. To identify hydrogen bonds, the HBPLUS program finds all proximal donor (D) and acceptor (A) atom pairs that satisfy specified geometrical criteria for bond formation. The criteria used for the current study were H-A distance < 2.7 Å, D-A distance < 3.35 Å, D-H-A angle > 90º and H-A-AA angle > 90º, where AA is the atom attached to the acceptor. A residue was defined to be in van der Waals contacts in the interface with the DNA if any of its atoms fell within a 3.9 Å cutoff distance from any of the DNA atoms in the Zif268-DNA complex.

**Table S2 (A):** Intermolecular hydrogen bonds calculated from the Zif268–DNA crystal structure by use of the HBPLUS program.

| Amino acid | Atom | Base | Atom | Distance (Å) |
| --- | --- | --- | --- | --- |
| Arg^70^ | NH2 | Gua^2^ | OP2 | 2.87 |
| Arg^80^ | NH2 | Gua^2^ | N7 | 2.86 |
| Arg^80^ | NH1 | Gua^2^ | O6 | 2.99 |
| His^53^ | ND1 | Gua^4^ | OP1 | 2.66 |
| Arg^74^ | NH1 | Gua^4^ | N7 | 2.97 |
| Arg^74^ | NH2 | Gua^4^ | O6 | 2.54 |
| Ser^45^ | OG | Gua^6^ | OP2 | 2.84 |
| His^49^ | NE2 | Gua^6^ | N7 | 2.81 |
| Arg^14^ | NE | Gua^7^ | OP1 | 2.48 |
| His^25^ | ND1 | Gua^7^ | OP2 | 2.86 |
| Arg^46^ | NH1 | Gua^7^ | N7 | 2.93 |
| Arg^46^ | NH2 | Gua^7^ | O6 | 2.85 |
| Arg^3^ | NH1 | Gua^8^ | OP1 | 2.90 |
| Arg^24^ | NH2 | Gua^8^ | N7 | 2.78 |
| Arg^24^ | NH1 | Gua^8^ | O6 | 2.93 |
| Arg^18^ | NH1 | Gua^10^ | N7 | 2.79 |
| Arg^48^ | NH2 | Gua^10^ | O6 | 2.67 |
| Asp^48^ | OD2 | Cyt^5^ | N4 | 3.00 |
| Ser^75^ | OG | Cyt^6^ | OP2 | 2.75 |

**Table S2 (B):** Intermolecular van der Waals contacts calculated from the Zif268–DNA crystal structure by use of the HBPLUS program.

| Amino acid | Atom | Base | Atom | Distance (Å) |
| --- | --- | --- | --- | --- |
| Glu^77^ | OE2 | Gua^2^ | C2' | 3.86 |
| Thr^56^ | CG2 | Cyt^3^ | P | 3.63 |
| Thr^56^ | CB | Cyt^3^ | OP1 | 3.38 |
| Thr^56^ | CG2 | Cyt^3^ | OP1 | 3.43 |
| Thr^56^ | CG2 | Cyt^3^ | OP2 | 3.82 |
| Thr^56^ | CG2 | Cyt^3^ | O5' | 3.31 |
| Arg^42^ | NH2 | Gua^4^ | C4' | 3.86 |
| Arg^42^ | NH2 | Gua^4^ | O3' | 3.78 |
| Arg^42^ | NH1 | Thy^5^ | P | 3.74 |
| Arg^42^ | NH1 | Thy^5^ | OP2 | 3.01 |
| Phe^44^ | CD1 | Thy^5^ | OP2 | 3.56 |
| Phe^44^ | CE1 | Thy^5^ | OP2 | 3.70 |
| Ile^28^ | CG2 | Gua^6^ | P | 3.90 |
| Ile^28^ | CG2 | Gua^6^ | OP1 | 3.54 |
| Ile^28^ | CG2 | Gua^6^ | O5' | 3.36 |
| Ile^28^ | CG2 | Gua^6^ | C5' | 3.74 |
| Phe^16^ | CD1 | Gua^8^ | OP2 | 3.60 |
| Phe^16^ | CE1 | Gua^8^ | OP2 | 3.81 |
| Glu^21^ | OE2 | Gua^8^ | C2' | 3.35 |
| Glu^21^ | OE2 | Gua^8^ | C8 | 3.74 |
| Glu^21^ | OE2 | Gua^9^ | C5 | 3.61 |
| Glu^21^ | OE2 | Gua^9^ | C6 | 3.73 |
| Ser^19^ | OG | Gua^1^ | C7 | 3.03 |
| Asp^20^ | N | Thy^1^ | C7 | 3.65 |
| Asp^20^ | CB | Thy^1^ | C7 | 3.55 |
| Asp^20^ | CG | Thy^1^ | C7 | 3.78 |
| Asp^20^ | OD1 | Thy^1^ | C7 | 3.56 |
| Asp^20^ | OD2 | Ade^2^ | N7 | 3.66 |
| Asp^20^ | OD2 | Ade^2^ | N6 | 3.23 |
| Asp^20^ | OD2 | Ade^3^ | N4 | 3.82 |
| Lys^79^ | CD | Cyt^7^ | OP2 | 3.64 |
| Asp^76^ | CG | Ade^8^ | N6 | 3.76 |
| Asp^76^ | OD2 | Ade^8^ | N6 | 3.19 |

**Table S3 -** AIR set used for docking of YY1, WT1 and Aart with their corresponding DNA

| Molecule | zinc finger 1 | zinc finger 2 | zinc finger 3 |
| --- | --- | --- | --- |
| YY1  DNA | S338, V346  C6, A30 | F368, I376  C9, A27 | T398, T406  T13, T242 |
| WT1  DNA | S51, R60  G17, G6 | D80, T88  C21, C3 | D110, R114  C24, G2 |
| Aart  DNA | H120, H124  G10, G8 | D147, H152  C18, G5 | D175, H180  A21, G2 |

| AIRs set | Molecule | zinc finger1 | zinc finger 2 | zinc finger 3 |
| --- | --- | --- | --- | --- |
| Pairwise | Sp1  DNA | H20, W27  G9, C6 | D49, R53  C17, G5 | D77, K81  C20, G2 |
| Non-pairwise | Sp1  DNA | K17 W27  G9, G10 C6 | S48 R53  C15,C16 G5 | R76 K81  C18,G19 G2 |

**Table S4 -** AIR sets used for docking of Sp1 with DNA

In the non-pairwise set, N terminal residues of each α helix such as K17, S48 and R76 have

a combination with two bases.

**Figure S1:**  Reported binding mode of Sp1-DNA complex [24].


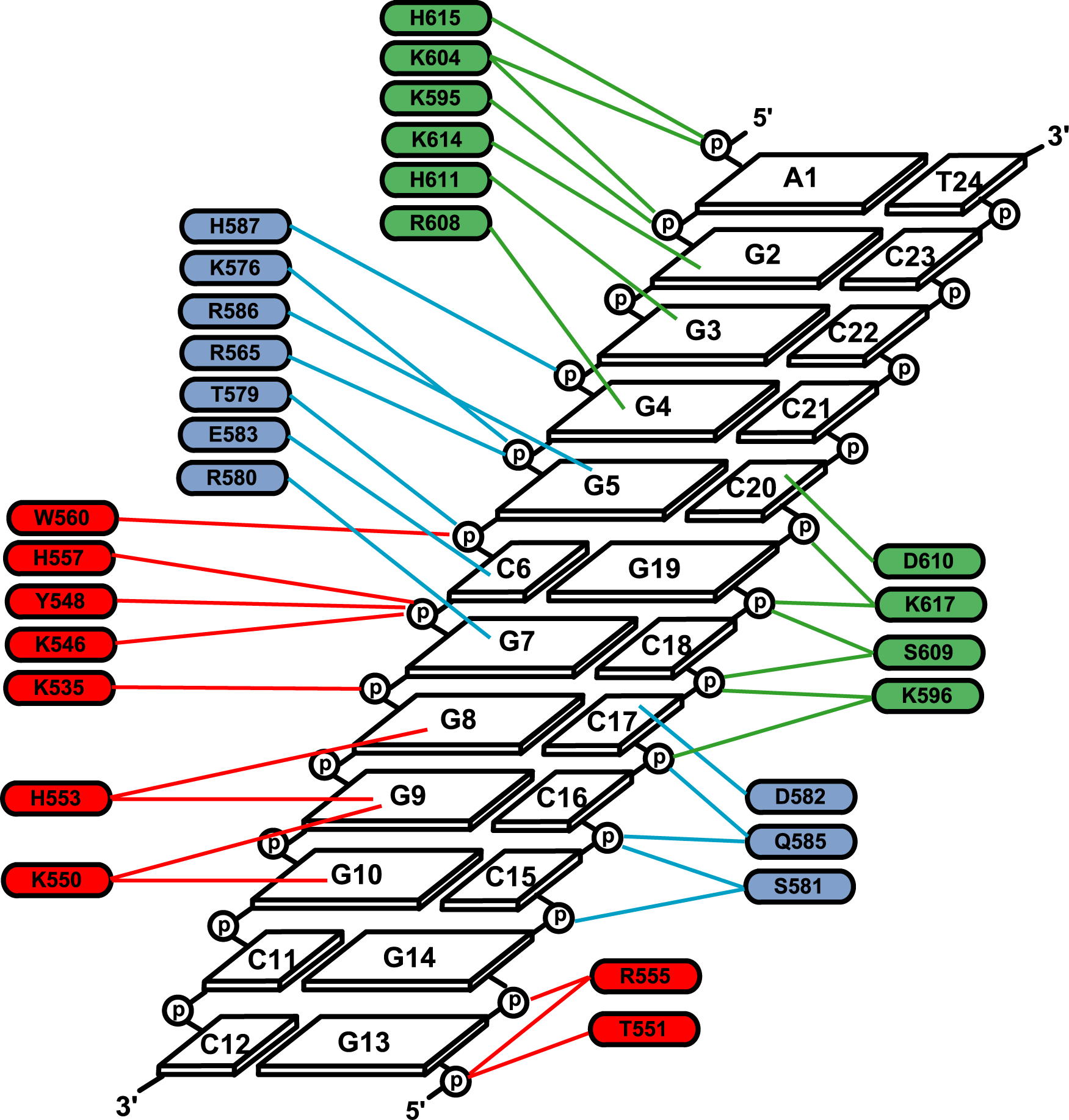


The interacting residues in zinc fingers 1, 2 and 3 are in red, blue and green respectively. The interacting DNA bases are represented as boxes. The phosphate backbones are represented as a chain of circles.

**Figure S2:**  Sequence of Zif268(A) and interacting DNA (B)

**
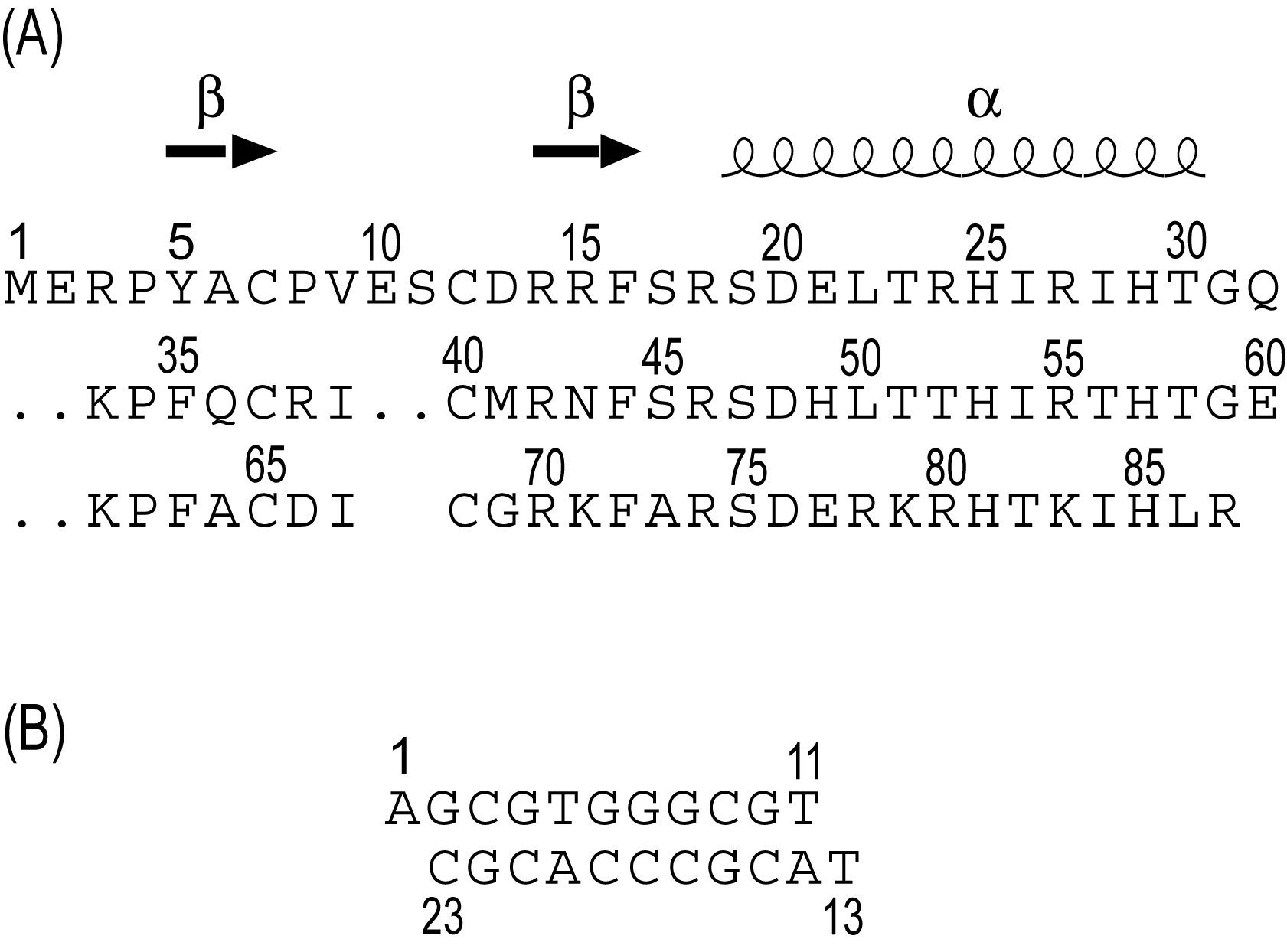
**
